# Acquired digital clubbing in 2 healthy young men: The incomplete form of pachydermoperiostosis (primary hypertrophic osteoarthropathy)

**DOI:** 10.1016/j.jdcr.2026.03.024

**Published:** 2026-03-19

**Authors:** Mary Kate Staunton, Mary Wu Chang

**Affiliations:** a University of Connecticut School of Medicine, Farmington, Connecticut; b Department of Dermatology, University of Connecticut School of Medicine, Farmington, Connecticut; c Department of Pediatrics, University of Connecticut School of Medicine, Farmington, Connecticut

**Keywords:** arthritis, clubbing, cutis verticis gyrata, pachyderma, pachydermia, periosteal thickening, periostosis, Schamroth sign, syndrome, systemic disease

## Introduction

Digital clubbing (bulbous deformity of the tips of the digits) is the oldest clinical sign in medicine, first described by Hippocrates around 450 BCE (aka “Hippocratic fingers” or Schamroth sign). It is classically associated with cardiopulmonary disease and is classified as secondary clubbing. In contrast, primary clubbing is much less common and under-recognized. Pachydermoperiostosis (PDP), also known as primary hypertrophic osteoarthropathy (HOA) is a rare genetic condition which causes primary digital clubbing without underlying systemic disease. We present 2 healthy young men who developed digital clubbing at 16-years and 23-years of age and were diagnosed with the incomplete form of PDP, also known as primary HOA.

## Case report

**Fig 1:**
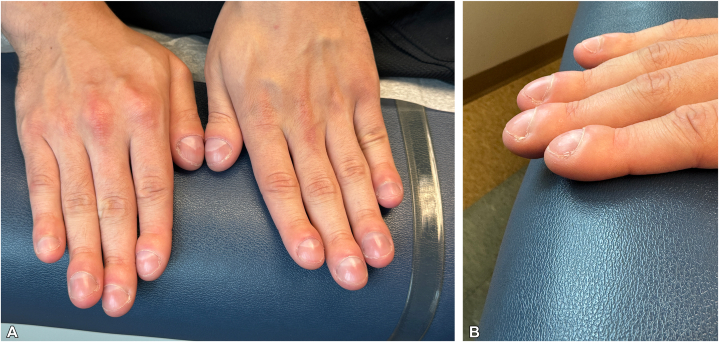
**A** and **B,** Bulbous deformity (clubbing) of the fingertips in Patient 1. These changes developed at age 16 years.

**Fig 2:**
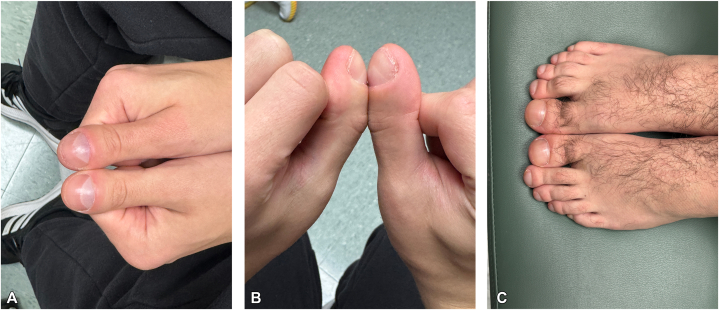
**A-C,** A healthy 30-year-old male (Patient 2) with asymptomatic clubbing of fingers and toes since age 23 years. **A,** Clubbing of thumbs. **B,** Note positive Schamroth sign (obliteration of the normal diamond shaped space between the fingers) **C,** Clubbing of toes.

Patient 1: A 16-year-old healthy male presented to the pediatric dermatology clinic for ongoing acne management. He mentioned new onset of “swelling” of the fingernails and toenails. The patient reported that this “swelling” arose spontaneously with no known triggers or contributing factors and progressed over the past year. The patient was healthy and active, did not smoke or vape, and denied pulmonary or cardiac symptoms. Family history was negative for clubbing, skin thickening, or cardiopulmonary issues. On examination, all fingers and toes exhibited digital clubbing with a positive Schamroth sign (Fig 1), but no pachyderma. A complete blood count, celiac testing, and chest x-ray were all within normal limits. Hand X-rays showed periosteal reaction along the shafts of the bilateral first metacarpal bones and the left distal radius, characteristic of PDP (Fig 2). Genetic testing was recommended but declined by the patient’s family. With no underlying systemic illness identified, the incomplete form of PDP was diagnosed (primary HOA). At 2-year follow-up the patient was healthy, but the digital clubbing had progressed, causing the patient to be embarrassed and hide his fingers.

**Fig 3:**
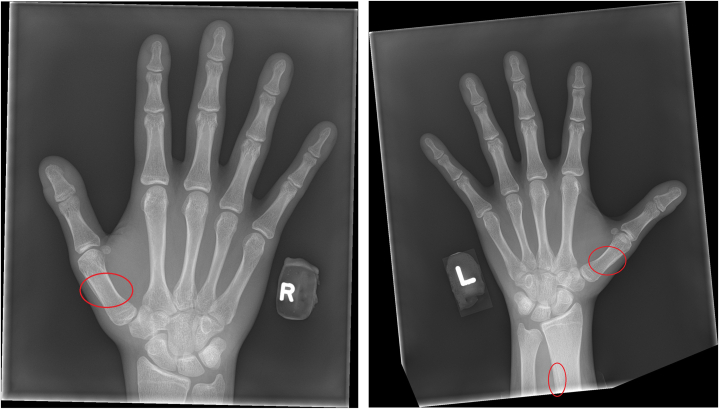
Xrays of the hands of Patient 1. Mild periosteal reactions along the shafts of the bilateral first metacarpal bones and the left distal radius, characteristic of PDP, are *circled*. *PDP*, Pachydermoperiostosis.

Patient 2: A 30-year-old healthy male was noted to have clubbing as an incidental finding. He reported he was diagnosed with primary HOA at age 23. He stated that he was evaluated after a peer had noticed swelling in his fingertips. The patient had not noticed the digital clubbing before that time and was unsure when it may have first appeared. The patient denied cardiopulmonary illness and did not smoke or vape. The patient reported mild acne and seborrheic dermatitis in adolescence, but no other skin disease. Family history was negative for clubbing, skin thickening, or cardiopulmonary issues. On physical examination, all fingers and toes exhibited digital clubbing with positive Schamroth sign (Fig 3). There was no pachyderma. Echocardiogram, cardiac magnetic resonance imaging, and chest X-rays were normal. Imaging of the hands and feet were not done, nor genetic testing. His team of physicians ruled out underlying systemic disease processes at that time and the diagnosis of primary HOA was made. The patient remained healthy, asymptomatic, and active at age 30 years and denied progression of the clubbing.

## Discussion

PDP is a rare genetic disease characterized by the triad of skin hypertrophy, digital clubbing, and periosteal proliferation of the bones. It presents in early childhood or at puberty and affects males at a ratio of 9:1.^1^^,^^2^ The complete form of PDP is characterized by all 3 features: pachyderma (skin hypertrophy causing furrowing of the forehead skin and cutis verticis gyrata of the scalp), digital clubbing, and periosteal proliferation of long bones. The incomplete form (also known as primary HOA) consists of digital clubbing and periosteal changes but lacks pachyderma. The 2 cases presented herein exemplify incomplete PDP or primary HOA. Finally, a third “form fruste” presents with predominant pachyderma and minimal periostosis.^3^

Other features of PDP include arthralgias, present in 20% to 40% of cases. It typically affects the knees, ankles, and wrist and may be accompanied by synovial effusion.^1^ Other skin manifestations include those related to glandular hypertrophy (seborrhea, blepharoptosis, acne, and hyperhidrosis.) Historically, severe cases were known as Touraine-Solente-Gole syndrome (coarsening of the facial features, nonpitting soft tissue swelling at the ankles, thickening of the ankles and wrists, and joint effusions).

Digital clubbing is a cardinal sign of PDP and usually the sole clinical manifestation, but thorough initial evaluation is needed to rule out secondary clubbing resulting from underlying systemic disease. Bone changes related to secondary HOA tend to develop more rapidly with less skin involvement.^4^ Pseudo-clubbing, with an angle of less than 180 between the nail plate and proximal nail fold, is associated with hyperparathyroidism, chronic kidney disease, scleroderma, and sarcoidosis. Thyroid acropachy may also present with digital clubbing and hypertrophic osteopathy.

Several theories have been proposed regarding the pathophysiology of PDP. Vascular endothelial growth factor induces vascular hyperplasia, new bone formation, and edema and has been implicated as a link between the various manifestations of the disease.^5^ With the rise of genetic testing, 2 main genes have been implicated in the pathogenesis of PDP—*HPGD*, which encodes a prostaglandin E2 (PGE2) catabolizing enzyme, and *SLCO2A1*, which encodes a transporter responsible for the uptake of PGE2; dysfunction of these genes are thought to cause elevated levels of PGE2.^6^^,^^7^ Furthermore, higher levels of PGE metabolites in the urine correlate with the complete form of PDP, suggesting it may be useful in distinguishing between phenotypes of the disease.^8^ Further research is needed to understand the relationship between PGE2 and vascular endothelial growth factor and their relation to disease progression.

The prognosis of the incomplete form of PDP (primary HOA) is good, it is usually a self-limiting disease and manifestations become stable or may resolve spontaneously. No treatment is available to improve the clubbing for any form of PDP. For progressive PDP, it has been postulated that NSAIDs could be used to block PGE2 synthesis, but more research is needed to verify effective therapy.^9^

## Conflicts of interest

None disclosed.
